# Systematic review of the prevalence of Gastrointestinal helminths in ruminants in Mexico

**DOI:** 10.1007/s11259-025-10968-6

**Published:** 2025-11-15

**Authors:** Roberto González-Garduño, Rosa Isabel Higuera-Piedrahita, Jorge Alfredo Cuéllar-Ordaz, Abel Villa-Mancera, Pedro Mendoza-de Gives, J. Felipe Torres-Acosta

**Affiliations:** 1 Unidad Regional Universitaria Sursureste, Universidad Autónoma Chapingo, Km 7.5, Carr. Teapa-Vicente Guerrero, Tabasco, C.P. 86800 México; 2 Facultad de Estudios Superiores Cuautitlán, Unidad de Investigación Multidisciplinaria, Laboratorio 3, Universidad Nacional Autónoma de México, Carretera Cuautitlán-Teoloyucan km 2.5, C.P. 54714 San Sebastián Xhala, Cuautitlán Izcalli México; 3 Facultad de Medicina Veterinaria y Zootecnia, Benemérita Universidad Autónoma de Puebla, Tecamachalco, C.P. 75460 Puebla Mexico; 4 Centro Nacional de Investigación Disciplinaria en Salud Animal e Inocuidad, Instituto Nacional de Investigaciones Forestales, Agrícolas y Pecuarias (INIFAP), Jiutepec, Carretera Federal Cuernavaca-Cuautla No. 8534, Col. Progreso, C.P. 62574 Morelos Mexico; 5 Facultad de Medicina Veterinaria y Zootecnia, Universidad Autónoma de Yucatán, C.P. 97203 Mérida, Yucatán México

**Keywords:** Cestodes, Gastrointestinal helmints, Identification, Nematodes, Tropic, Systematic review, Prevalence, Mexico

## Abstract

In Mexico, the abundance of endoparasites that affect the health of ruminants and the economy of the farmer, so the objective of this review was to determine the prevalence of the main genera and species of ruminant helminths that have been identified in Mexico in the last 37 years. A systematic search was carried out following the PRISMA 2020 guidelines and supported by the Elsevier platform (Scopus and ScienceDirect), Google Scholar, Redalyc and Scielo tools. In addition, information was sought in the proceedings Congress of the Buiatrics and Veterinary Parasitologists. A database was created with 36 documents containing information on the prevalence of gastrointestinal parasites in Mexico. Research studies that included treatments that affected prevalence were not included, nor were those of an experimental nature that did not aim to study prevalence. The prevalence for *Haemonchus contortus*, the main abomasal nematode in ruminants, was 38.8%. In the case of *Mecistocirrus*, only four studies indicated the prevalence in cattle, which was estimated at 40.5%. In the small intestine, the most prevalent nematode in cattle were *Cooperia* and *Strongyloides*. In goats and sheep, the presence of *Trichostrongylus*, and *Toxocara* were indicated. The main cecal nematodes include *Trichuris* and in the colon *Oesophagostomum* and *Chabertia*. The high prevalence of gastrointestinal nematodes in ruminants makes it essential to consider control measures to reduce their prevalence.

## Introduction

Identification of the genus and species of gastrointestinal parasites (GIP) affecting ruminants help to establish appropriate measures for parasite control on farms. In addition to coproparasitoscopic diagnosis, identification of adult parasites can be performed after necropsy of dead or slaughtered animals (González-Garduño et al. 2014). To obtain a diagnosis of the gastrointestinal nematodes (GIN) species involved, eggs are initially detected using a flotation technique or quantified using the McMaster technique (Thienpont et al. 2003; Cringoli et al. 2004), of which there are currently a number of variants such as the FLOTAC technique (Cringoli et al. 2010) and FECPAK^G2^ (Boelow et al. 2022). However, these techniques are limited in that they generally identify only the parasite family or, in some cases, the genus—but rarely allow for species-level identification among(Thienpont et al. 2003), therefore, one possibility for accurate diagnosis is the identification of infective larvae obtained from larval cultures to determine the genus and in some cases the species (van Wyk and Mayhew 2013). But when studies require the identification of parasites present in the infectious process, post-mortem studies must be performed that allow the recovery of adult parasites (López Ruvalcaba et al. 2013) and thus direct parasite control programs according to the species present in a region. Another possibility is the identification of the species through molecular biology techniques that are available in many research institutions (Amarante et al. 2017). In small ruminants, the importance of diagnosing gastrointestinal nematodes is essential because there are some that endanger the lives of animals. One of the most important is *Haemonchus contortus*, a highly prevalent and virulent species in sheep and goats. Its blood-feeding behavior often results in severe anemia, physical deterioration, and death in heavily infected or susceptible animals. In Mexico, climatic diversity causes an abundance of endoparasites that affect the health of ruminants and the economy of the farmer, so the objective of this review is to determine the prevalence of the main genera and species of ruminant helminths that have been identified in Mexico in the last 37 years.

The importance of diagnosing gastrointestinal nematodes (GINs) is paramount in small ruminants, as some species not only compromise productivity but also endanger the lives of animals. Among the most pathogenic is *Haemonchus contortus*, a blood-sucking nematode of the abomasum with high prevalence and virulence in sheep and goats. Its blood-feeding habit leads to severe anemia, hypoproteinemia, edema, and death in susceptible animals, particularly under conditions of high parasitic load (Besier et al. 2016; Adduci et al. 2022a, b; Kapó et al. 2024). Beyond its impact on animal health, haemonchosis is a primary constraint for goat production in regions such as East Africa (Githiori et al. 2006; Perry et al. 2002), and it is the most prevalent GIN in countries like Uganda (Nsereko et al. 2015; Kalule et al. 2023). Other GINs, such as species of the genus *Trichostrongylus*, also have a significant economic impact by affecting appetite and nutrient absorption or utilization in the small intestine (Francis et al. 2020). Furthermore, some *Trichostrongylus* species are of zoonotic importance, with a broad geographical distribution, particularly in the Middle East, Far East, and some African regions (Abbas and Hildreth 2022; Ghasemikhah et al. 2011). A high prevalence and diversity of trichostrongylid species in ruminants has been reported, as observed in northern Iran, where the total infection prevalence was 74.6% in sheep and 84.6% in goats (Hosseinnezhad et al. 2021). The control of these parasites is further complicated by the emerging issue of anthelmintic resistance, particularly to benzimidazoles, as genetically documented in *H. contortus* via mutations in the β-tubulin gene in countries like Uganda and Sudan (Kalule et al. 2023; Mohammedsalih et al. 2020).”

## Materials and methods

**Fig. 1:**
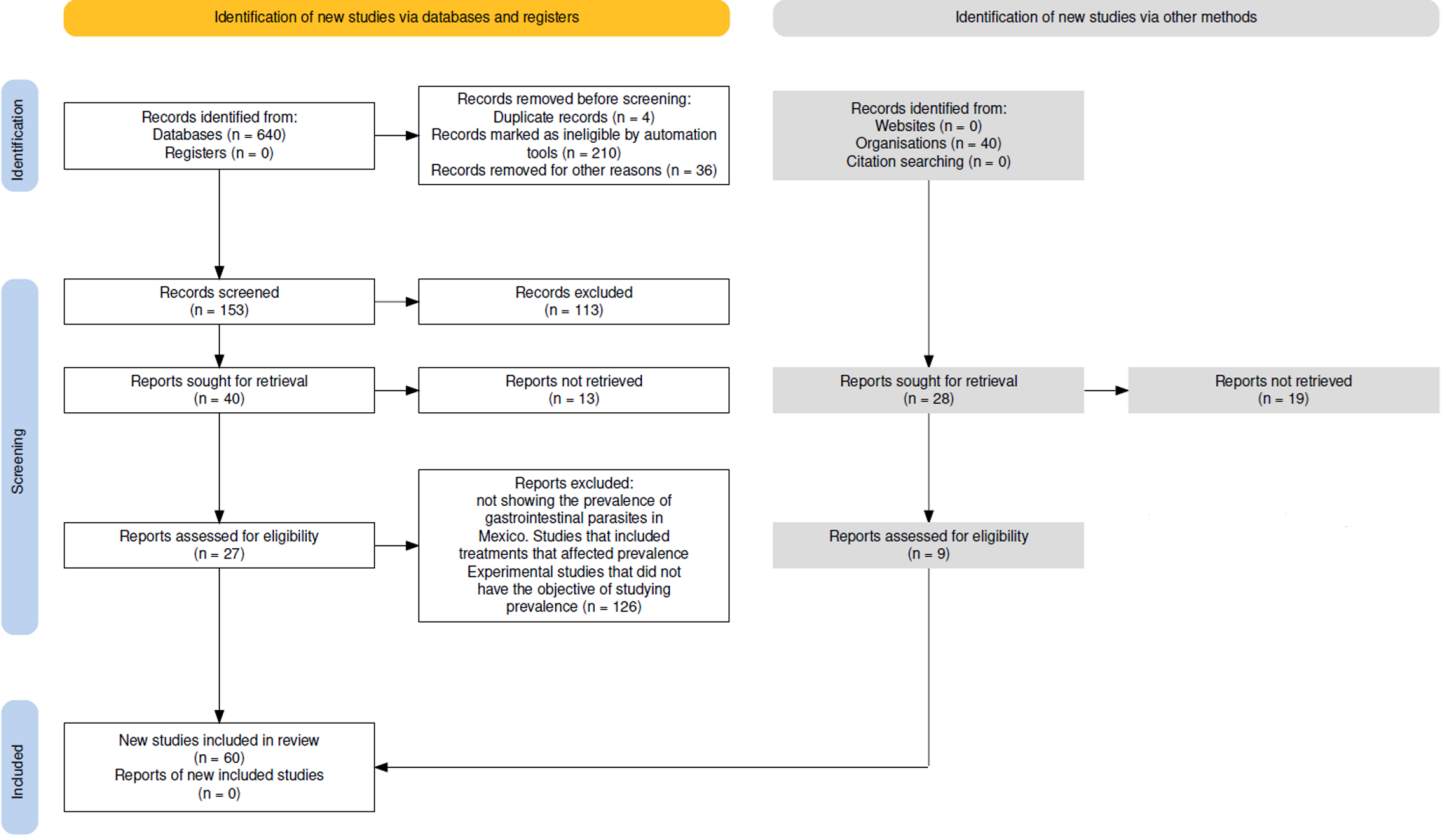
Flow diagram for the search for information on the prevalence of helminths in Mexico according to PRISMA

To carry out this study, a systematic search was carried out following the procedure using the flow diagram (Fig. 1) of the Preferred Reporting Items for Systematic reviews and Meta-Analyses (PRISMA) 2020 website (Page et al. 2021).

Manuscripts were searched using the Elsevier platform (Scopus and ScienceDirect), Clarivate (Web of Science), Google Scholar, Redalyc and Scielo. Information was also sought in the proceedings of the congresses of the Mexican Association of Veterinarian Specializing in Bovines AC (Buiatría 2003, 2012, 2014, 2015, 2019, 2022) and the Mexican Association of Veterinary Parasitologists (AMPAVE 2012, 2017, 2019). Of 640 records displayed in the search engines that included articles, theses, books, meta-analyses and other references, only 36 were cited with information on the prevalence of gastrointestinal parasites in Mexico. Previously, 153 reviewed manuscripts were related to veterinary parasitology topics in ruminants, of which 113 were discarded as they did not indicate the prevalence of gastrointestinal parasites in Mexico and only the documents that addressed the prevalence of the main gastrointestinal nematodes (*Haemonchus*,* Ostertagia*,* Teladorsagia*,* Cooperia*), which infect ruminants in Mexico, were selected, so the scientific names were used as keywords and searched in English and Spanish, in addition the following words were used combined with the scientific names: prevalence, frequency, gastrointestinal nematodes, small ruminants, sheep, goats, buffalos, cows, cattle and the word Mexico was included in all searches. Research studies that included treatments with which the prevalences were affected or of experimental nature that did not have the objective of studying the prevalence were not included in the review (Table 1).

**Table 1:** Gastrointestinal nematodes that parasitize the abomasum, small intestine, cecum and colon of cattle, sheep and goats according to the authors of prevalence studies in Mexico

	Sample origin		Abomasum			Small intestine					Cecum and colon			
Species		*N*	Hac	Ost	Mec	Coo	Tric	Stron	Bun	Tox	Oes	Cha	Trich	Autor
Cattle	Slaughterhouse	100			Ӂ	Ӝ				Ӝ	֍			(Mejía García and Orozco de Gortari 1987)
Sheep	Fecal	40	Ӂ			Ӝ	Ӝ	Ӝ	Ӝ		֍			(George Sánchez and Quiroz Romero 1993)
Cattle	Fecal	1908	Ӂ	Ӂ		Ӝ	Ӝ	Ӝ	Ӝ	Ӝ	֍		֍	(Domínguez Alpízar et al. 1993)
Ruminants	Fecal	3827						Ӝ					֍	(Rodríguez-Vivas et al. 2001)
Sheep	Fecal	312	Ӂ				Ӝ				֍			(Nahed-Toral et al. 2003)
Goats	Fecal	132	Ӂ	Ӂ		Ӝ	Ӝ	Ӝ			֍	֍	֍	(Avelino et al. 2003)
Goats	Fecal	Na	Ӂ	Ӂ		Ӝ	Ӝ	Ӝ		Ӝ				(Aguilar et al. 2003)
Goats	Fecal	8	Ӂ				Ӝ	Ӝ			֍			(Torres-Acosta et al. 2004)
Cattle	Slaughterhouse	5	Ӂ			Ӝ			Ӝ					(Vásquez-Prats et al. 2004)
Cattle	Fecal	336	Ӂ			Ӝ	Ӝ				֍			(Olivares-Pérez et al. 2006)
Sheep	Fecal	219	Ӂ			Ӝ	Ӝ	Ӝ			֍		֍	(Rojas-Hernández et al. 2007)
Sheep	Slaughterhouse	242	Ӂ			Ӝ	Ӝ	Ӝ	Ӝ		֍		֍	(González-Garduño et al. 2011)
Goats	Slaughterhouse	10		Ӂ		Ӝ	Ӝ		Ӝ		֍			(Olivares-Perez et al. 2012)
Sheep	Grasslands	30	Ӂ			Ӝ	Ӝ							(Flota-Bañuelos et al. 2013)
Sheep	Fecal	50	Ӂ	Ӂ		Ӝ	Ӝ	Ӝ	Ӝ		֍			(Acevedo-Ramírez et al. 2013)
Cattle	Slaughterhouse	68	Ӂ		Ӂ									(von Son-de Fernex et al. 2014)
Cattle	Fecal	214	Ӂ	Ӂ		Ӝ	Ӝ			Ӝ		֍	֍	(Felipe et al. 2014)
Cattle	Fecal	50	Ӂ			Ӝ		Ӝ						(Landeros et al. 2014)
Sheep	Fecal	29	Ӂ	Ӂ		Ӝ	Ӝ		Ӝ		֍	֍		(Acevedo et al. 2014)
Cattle	Fecal	214	Ӂ			Ӝ	Ӝ			Ӝ		֍	֍	(Fernández-Figueroa et al. 2015)
Sheep	Grasslands	117	Ӂ	Ӂ		Ӝ	Ӝ	Ӝ		Ӝ	֍	֍	֍	(Sánchez Brito 2017)
Buffaloes	Fecal	383						Ӝ					֍	(Ojeda-Robertos et al. 2017)
Sheep	Fecal	90	Ӂ	Ӂ		Ӝ	Ӝ				֍	֍		(Acevedo-Ramírez et al. 2017)
Cattle	Fecal	100	Ӂ			Ӝ	Ӝ				֍			(Ortiz-Timoteo et al. 2017)
Goats	Fecal	101		Ӂ		Ӝ	Ӝ							(Figueroa Antonio et al. 2018)
Goats	Fecal	371	Ӂ	Ӂ			Ӝ							(Olivas-Salazar et al. 2018)
Goats	Slaughterhouse	499	Ӂ			Ӝ	Ӝ	Ӝ			֍			(Munguía-Xóchihua et al. 2018)
Goats	Fecal	262						Ӝ					֍	(Barrón et al. 2019)
Sheep	Fecal	177	Ӂ	Ӂ		Ӝ	Ӝ				֍	֍		(Castellanos et al. 2019)
Cattle	Fecal	411	Ӂ	Ӂ		Ӝ			Ӝ					(Castillo et al. 2019)
Cattle	Fecal	32						Ӝ		Ӝ			֍	(Hernández et al. 2019)
Sheep	Fecal	Na	Ӂ				Ӝ	Ӝ						(Pérez et al. 2019)
Sheep	Fecal	216	Ӂ			Ӝ	Ӝ	Ӝ			֍			(Camacho Ronquillo et al. 2021)
Cattle	Fecal	185						Ӝ			֍			(Ortiz-Muñoz et al. 2021)
Sheep	Fecal	300	Ӂ			Ӝ	Ӝ	Ӝ	Ӝ					(García et al. 2022)
Cattle	Fecal	190	Ӂ	Ӂ		Ӝ					֍	֍		(Salazar et al. 2022)
Sheep	Fecal	126	Ӂ			Ӝ	Ӝ				֍			(Valladares-Carranza et al. 2024)
Total authors			**28**	**13**	**2**	**27**	**26**	**19**	**9**	**7**	**21**	**8**	**11**	

*N* Number of samples. *Hac* *Haemonchus*, *Ost* *Ostertagia*, *Mec* *Mecistocirrus*, *Coo* *Cooperia*, *Tric* *Trichostrongylus*, *Stron* *Strongyloides*, *Bon* *Bunostomum*, *Tox* *Toxocara*, *Oes* *Oesophagostomum*, *Cha* *Chabertia*, *Trich* Trichuris. *Na* not available

For the analysis of the information, the JASP program (JASP Team, 2024) was used to obtain measures of central tendency and dispersion from the database formed in EXCEL, in which the prevalence averages were obtained and analyzed together with independent variables such as the species of ruminant, the type of study (fecal, slaughterhouse or pasture), the state of the republic and the month of the year, so the model used was analyzed in SAS (SAS 2017):

$$y_{ijklm}=\mu+\gamma_i+\gamma\tau_{i\left(j\right)}+\delta_k+\theta_1+\varepsilon_{ijklm}$$

where $\:{\boldsymbol{y}}_{\boldsymbol{i}\boldsymbol{j}\boldsymbol{k}\boldsymbol{l}}=$ Prevalence, **µ =** general mean, $\:{\boldsymbol{\gamma\:}}_{\boldsymbol{i}}$= effect of the i-th specie (Buffalo, Cattle, Sheep, Goats), $\:{\boldsymbol{\tau\:}}_{\boldsymbol{i}\left(\boldsymbol{j}\right)}$= effect of sample type nested in the i-th specie (fecal, slaughterhouse or pasture), $\:{\boldsymbol{\delta\:}}_{\boldsymbol{k}}$= effect of k-th state of the republic (1,2,3, … 14), **θ**_**l**_ = effect of l-th month of year (l=January, February, … December), and $\varepsilon$_**ijklm**_**=** experimental error.

## Results

The study of gastrointestinal parasites in Mexico for 37 years (1987–2024) has considered Platyhelminthes and roundworms (Table 2) as the main agents that affect the health of ruminants, although there are other studies in which protozoa are also described as highly prevalent in Mexico (García et al. 2022; Valladares-Carranza et al. 2024). However, this review focuses primarily on nematodes; however, due to its high prevalence, the cestode Moniezia is also included.

**Table 2:** Prevalence (%) of Gastrointestinal nematodes in Mexico, according to sample type collected

	Haemonchus				Teladorsagia/Ostertagia				Mecistocirrus	
Sample type	N.	N2	Prevalence	SD	N.	N2	Prevalence	SD	N.	Prevalence
Cattle										
Eggs/Larvae (Feces)	7	16	33.4	23.8	5	5	26.4	41.3		
Slaughterhouse(worms)	2	5	16.0	10.3	1	1	9.6	-	2	40.5
Goats										
Eggs/Larvae (Feces)	2	5	20.8	19.9		6	4.3	3.9		
Slaughterhouse(worms)	2	7	63.6	21.9	1	2	12.0	11.3		
Sheep										
Eggs/Larvae (Feces)	10	23	50.2	25.0	4	8	7.8	6.7		
Slaughterhouse(worms)	1	1	37.2	.						
Larvae (Pasture)	3	22	50.3	38.4	2	3	35.0	31.4		

*N* Number or authors, *N2* number of records, *SD* Standard deviation

The taxonomic classification of the prevalent nematodes in Mexico has been taken from a recent study of the classification of the Phylum Nematoda Cobb, 1932 (Hodda 2022) Therefore the order and family of each of them is listed below:

- Order **Rhabditida**, Family Trichostrongylidae Leiper, 1908: *Haemonchus*,* Cooperia*,* Trichostrongylus*,* Mecistocirrus. Ostertagia*,* Teladorsagia*.
- Order **Rhabditida**. Family Strongylidae Baird, 1853. *Oesophagostomum*,* Chabertia*.
- Order **Rhabditida** Family Ancylostomatidae Looss, 1905. *Bunostoth highmum*.
- Order **Panagrolaimida**. Family Strongyloididae Chitwood & MacIntosh, 1934. *Strongyloides*.
- Order **Trichocephalida**. Family Trichuridae Ransom, 1911. *Trichuris*.
- Order **Spirurida**, Family Ascarididae Baird, 1853. *Toxocara*.

### Abomasal nematodes

The genus *Haemonchus* Cobb 1898, is the most studied due to its economic and veterinary importance. Many publications have been dedicated to describing this genus, such as the book edited by Gasser and Von Samsom-Himmelstjerna (Gasser and Von Samson-Himmelstjerna 2016) and many other authors who have described it in sheep (Flay et al. 2022; Mohamed et al. 2024), cattle (Jabbar et al. 2014; Haider et al. 2024), goat (Adduci et al. 2022a, b; Kapo et al. 2024) y South American camelids (Jabbar et al. 2013; Zahid et al. 2024). Of the 14 species currently recognized (Hodda 2022), *Haemonchus contortus* has been described as an abomasal parasite in sheep and other ruminants (Zarlenga et al. 2016). *H. contortus* and *Haemonchus placei* are also known as barber pole worms. They are the two main species of this parasite that is found in small ruminants and cattle, respectively, however, hybrids produced by crossing *H. placei* and *H. contortus* can be found, although they have low fertility or are sterile due to meiotic abnormalities (Amarante et al. 2017).

Another genus of abomasal nematode is *Mecistocirrus* Railliet & Henry, 1912 (Hodda 2022) with four species, which has a high prevalence in southeastern Mexico and has been little studied. Since 1965, there has been extensive knowledge of the life cycle of *M. digitatus* and of larval development and anatomical aspects of larvae and adults (Fernando 1965). In Mexico the discovery was reported for the first time in 1987 (Mejía García and Orozco de Gortari 1987), in that same year the third stage larva of the parasite was described(García Ortiz and Mejía García 1987) and since then only one study indicated that *M. digitatus* was present in the humid tropical regions but they did not report its prevalence (Vásquez-Prats et al. 2004). Later, in 2013, a morphological description of this species observed in the state of Tabasco and Chiapas was made (González-Garduño et al. 2013). In the following year its presence was also indicated, and the identification was made by electron microscopy and by PCR in the Mexican tropics (von Son-de Fernex et al. 2014). In 2017, *M. digitatus* was identified from one animal at necropsy in a study group treated with netobimin under an organic milk production system (Ortiz Pérez et al. 2017).

In the subtribu Ostertaginii Skryabin & Schulz, 1937 (Hodda 2022), 10 species have been reported to be present in cattle, sheep and goats in North America. The species described include *Ostertagia ostertagi* and *O. lyrata* in cattle and *Teladorsagia circumcincta*, *T. trifurcata* and *T. davtiani* in sheep and goats with a worldwide distribution and are the main causative agents of ostertagiosis (Lichtenfels et al. 1988). Polymorphism has also been described among males, generating identification keys for nine species (Lichtenfels and Hoberg 1993). In addition, a comprehensive review has been conducted including life cycle characteristics and implantation rate of *Ostertagia* (Verschave et al. 2014). In recent studies, the identification of nematodes of the *Teladorsagia* genus from ruminants has been carried out with the help of species-specific markers based on ITS2 rDNA, with which it is possible to accurately detect eggs and larvae of this genus (Ibrokhimov et al. 2023). In Mexico, *O. ostertagi* was reported in a slaughterhouse study (González-Garduño et al. 2011) and more recently the importance and prevalence of Ostertagia has been addressed in cattle in southern Mexico (Villa-Mancera et al. 2018).

### Prevalence of abomasal nematodes in Mexico

The average prevalence for *H. contortus*, the main abomasal nematode of ruminants in the different regions of Mexico was 38.8% (Table 2), mentioned by at least 28 authors. The prevalence by identifying larvae obtained by fecal cultures has been estimated at 40%. Similar values ​​have been observed in studies with slaughtered animals obtaining adult parasites (43%). While studies using the recovery of larvae in pastures contaminated with GIN during grazing have shown a 50% prevalence.

In the case of the brown abomasal worm or *Teladorsagia*/*Ostertagia*, the values ​​found in the literature indicate that cattle have the highest prevalence with identification of larvae and only one study indicates the recovery of larvae in pasture. In cattle and goats, the presence of *Teladorsagia*/*Ostertagia* identified in adults is reported. In the case of *Mecistocirrus*, only four studies indicate the prevalence of *Mecistocirrus* in cattle (Mejía García and Orozco de Gortari 1987; von Son-de Fernex et al. 2014), which was estimated at 40.5% (Table 2), in another study in the tropics of Mexico the presence of this nematode is also indicated, but without mentioning its prevalence (González-Garduño et al. 2013). On the other hand, in *Ovis canadensis*, a prevalence of 10 to 20% of *M. digitatus* has been indicated (García et al. 2022).

The distribution of *H. contortus* in cattle, sheep and goats in Mexico had the highest prevalence in southeastern states (51.4, 52.3 and 55.7% respectively). In the central states of Mexico, the prevalence in cattle and sheep is in second place (19.2 and 39.9% respectively) and only in the north of the country, the prevalence of this species in goats is in second place (29.6%) at the national level. It can also be noted that the largest number of studies have been carried out in sheep in which the prevalence is 39.7% and the lowest number of studies has been in goats with a prevalence of 35.9% (Table 3).

**Table 3:** Prevalence (%) of *Haemonchus contortus* by ruminant species and state of the Mexican Republic

State	Cattle			Goat			Sheep		
	*N*	Mean	SD	*N*	Mean	SD	*N*	Mean	SD
Estado de México	1	19.2					2	39.6	13.4
Morelos							20	40.3	30.7
Puebla				2	8.6	9.0			
Tlaxcala							4	40.0	9.2
Coahuila				1	4.0		1	2.0	
Nayarit	2	26.0	8.5						
Sinaloa	2	17.2	6.8				1	5.6	
Sonora	2	4.9	2.9	3	55.2	6.8			
Chiapas							1	80.0	
Guerrero	1	85.1		4	70.0	28.3	1	32.0	
Tabasco							1	37.2	
Veracruz	10	29.5	19.2				11	87.8	11.9
Yucatán	3	39.6	28.3	2	41.5	8.1			
Sureste							4	33.0	4.6
Average		31.7			35.9			39.7	

*N* Number of studies, *SD* Standard deviation

The prevalence of *Teladorsagia*/*Ostertagia* in cattle was 6.6 to 13% in Sonora, Veracruz and Yucatan and exceptionally in the State of Mexico a study indicates a 100% prevalence (Castillo et al. 2019). In goats, these nematodes were reported in Guerrero and Puebla with 4 to 8% prevalence, while in sheep the highest prevalence was 35.5% in the State of Mexico, while in Morelos, Sinaloa and Veracruz the maximum values ​​reached were 13%.

**Fig. 2:**
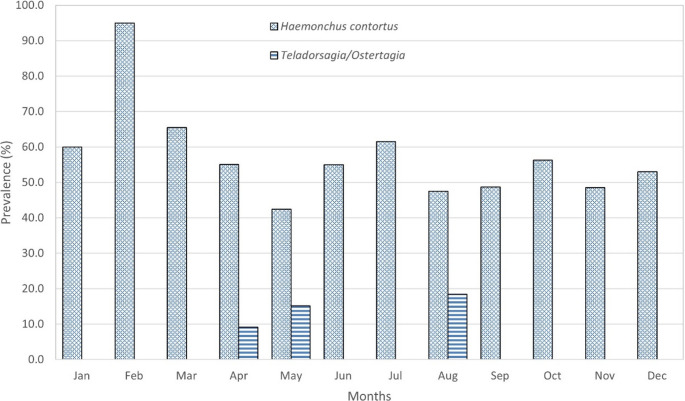
Monthly prevalence of *Haemonchus contortus* and *Teladorsagia*/*Ostertagia* in Mexico

The distribution of *H. contortus* throughout all months of the year indicates that this species has a wide distribution without seasonality and with consistently high prevalence, likely attributable to its prolific reproductive rate, which can reach up to 1,295 eggs per female per day (Saccareau et al. 2017) (Fig. 2).

### Nematodes of the small intestine

#### *Cooperia*

The genus *Cooperia* Ransom, 1907 has 33 species (Hodda 2022). In America, *Cooperia curticei* is the main species in sheep, while in temperate climates is *C. oncophora*, and the tropical species *C. pectinata* and *C. punctata* are the ones that parasitize and affect the health of cattle (Stewart 1954; Francis et al. 2020). These nematodes have a detrimental effect on both appetite and nutrient absorption or utilization by residing in the small intestine. Differentiation between *C. pectinata* and *C. punctata* is not possible in larvae, and it is very difficult to differentiate them from *C. oncophora*; the only reliable identification of a species is achieved by morphological analysis of adults, but this requires post-mortem analysis of the small intestine and is restricted to research settings (Francis et al. 2020), Therefore, molecular identification studies are more reliable. In Tabasco, Mexico, the presence of *C. curticei* and *C. punctata* was reported in 2013 and morphologically described in the small intestine of sheep and cattle, respectively (González-Garduño et al. 2014). Work has also been carried out on resistance to natural infection by *Cooperia* spp. in young Zebu x Holstein crossbred cattle in the tropics (García-Ruíz et al. 2019).

#### *Trichostrongylus*

The genus *Trichostrongylus* Looss, 1905 has 53 species (Hodda 2022), it is one of the most important zoonotic nematodes with wide geographic distribution in the world (Ghasemikhah et al. 2011; Abbas and Hildreth 2022). *Trichostrongylus* was widely described during 1932 and its presence in humans and ruminants is highlighted. The main species described were: *T. retortaeformis* Looss, 1905. *T. colubriformis* Ransom, 1911. *T. capricola* Ransom, 1907. *T. axei* Railliet and Henry, 1909. *T. vitrinus* Looss, 1905. *T. falculatus* Ransom, 1911. *T. rugatus* Monnig, 1925. *T. tenuis* Shipley, 1909. *T. pergracilis* Shipley, 1909. *T. affinis* Graybill, 1924. *T. calcaratus* Ransom, 1911. *T. probolurus* Looss, 1905. *T. orientalis* Jimbo, 1914. Due to the large number of *Trichostrongylus* species, molecular identification has become important, and a large number of studies have been published that identify this species molecularly (Zarlenga et al. 2001; Bakooie Katrimi et al. 2022).

#### *Strongyloides*

The genus *Strongyloides* Grassi, 1879 has 63 species (Hodda 2022). Although *Strongyloides* have been reported in different species of animals including ruminants and pigs and even birds and amphibians, the discovery was in humans with *S. stercoralis*. A broad description of this genus was made in 1925 (Sandground 1925) and later a study carried out in 1966 (Little 1966) established the criteria for the identification of *Strongyloides* describing parasitic species such as *S. stercoralis*, *S. fiilleborni*,* S. cebus*,* S. myopotami*,* S. venezuelensis* y *S. ratti*. For the determination of *Strongyloides*, PCR has currently been used (Dorris et al. 2002; Kramme et al. 2011; Saugar et al. 2015).

#### Prevalence of small intestine nematodes in Mexico

Of the small intestine nematodes in Mexico, those with the highest prevalence in cattle were *Cooperia* and *Strongyloides*, which were also mentioned in the largest number of records. In buffaloes, *Strongyloides* was also the nematode with the highest prevalence and the most reported. In goats and sheep, the nematode with the highest prevalence was *Trichostrongylus*, although *Toxocara* had a prevalence of 67% in a single study in goats, while in sheep only the reported prevalence was 6% (Table 4).

**Table 4:** Prevalence (%) of the main genera of small intestine helminths in ruminants in Mexico

	Cattle			Buffalos			Goat			Sheep		
**Genus**	N	Mean	SD	N	Mean	SD	N	Mean	SD	N	Mean	SD
*Cooperia*	17	46.2	5.5				10	21.0	7.6	34	8.0	1.8
*Trichostrongylus*	13	9.7	3.6				15	54.6	8.4	48	30.3	4.0
*Strongyloides*	21	16.6	2.5	4	41.8	6.9	7	19.0	3.7	18	14.9	4.9
*Bunostomun*	7	4.5	2.5				1	10.0	.	21	9.4	2.4
*Toxocara*	4	2.0	0.9	1	3.9	.	1	67.4	.	1	6.1	.
*Moniezia*	13	5.7	1.1	3	19.2	4.8	9	9.4	4.9	6	14.6	6.0

*N* Number of studies, *SD* Standard deviation

According to the stage at which prevalence was determined, *Trichostrongylus* was the GIN with the highest prevalence identified as an adult parasite obtained in a slaughterhouse. It was followed by *Cooperia*, and only one study indicated the presence of *Strongyloides* in a slaughterhouse (Table 5). The larvae recovered from copro-cultures corresponded to the highest prevalence of *Cooperia* in cattle and *Trichostrongylus* in goats. Only in sheep a prevalence study recovering larvae in pasture.

**Table 5:** Prevalence (%) of the three main genera of gastrointestinal nematodes of the small intestine in ruminants in Mexico

Species and sample type	*Cooperia*				*Trichostrongylus*				*Strongyloides*			
	N.	N2	Prevalence	SD	N.	N2	Prevalence	SD	N.	N2	Prevalence	SD
Buffalos												
Eggs/Larvae (Feces)										4	41.8	13.9
Cattle												
Eggs/Larvae (Feces)	8	16	45.6	23.1	6	13	9.7	12.8	6	21	16.6	11.7
Slaughterhouse (worms)	1	1	57.0									
Goats												
Eggs/Larvae (Feces)	3	6	7.8	7.5	4	11	42.6	29.8	5	7	19.0	9.8
Slaughterhouse (worms)	1	4	40.8	27.8	1	4	87.5	5.0				
Sheep												
Eggs/Larvae (Feces)	8	18	8.1	10.7	11	24	21.5	19.3	7	15	15.8	22.4
Slaughterhouse (worms)	1	1	36.0		1	1	25.2		1	1	3.3	
Larvae (Pasture)	3	15	5.9	7.9	3	23	39.8	32.5	2	2	14.3	17.6
Average			**28.7**				**37.7**				**13.8**	

*N* Number or authors, *N2* number of records, *SD* Standard deviation

In the genera with the lowest prevalence, only in one case a high prevalence value was observed in *Toxocara* in goats with 67%, while the second highest value corresponded to the cestode *Moniezia expansa* in sheep (Table 6).

**Table 6:** Prevalence (%) of the genera of gastrointestinal parasites of the small intestine with lower prevalence in ruminants in Mexico

	*Bunostomum*				*Toxocara*				*Moniezia*			
Species and sample type	N.	N2	Prevalence	SD	N.	N2	Prevalence	SD	N.	N2	Prevalence	SD
Buffalos												
Eggs/Larvae (Feces)					1	1	3.9		1	3	19.2	8.4
Cattle												
Eggs/Larvae (Feces)	2	2	10.3	12.6	3	3	2.4	2.0	6	11	6.6	3.7
Slaughterhouse(worms)	2	5	2.2	1.8	1	1	1.0		2	2	1.1	1.3
Goats												
Eggs/Larvae (Feces)					1	1	67.4		5	9	9.4	14.7
Slaughterhouse(worms)	1	1	10.0									
Sheep												
Eggs/Larvae (Feces)	5	12	3.2	4.1					3	4	10.3	9.2
Slaughterhouse(worms)	1	1	0.8						1	1	6.2	
Larvae (Pasture)	1	8	19.9	10.3	1	1	6.1		1	1	40.4	
Average	**7.7**				**19.2**				**12.3**			

*N* Number or authors, *N2* number of records, *SD* Standard deviation

**Fig. 3:**
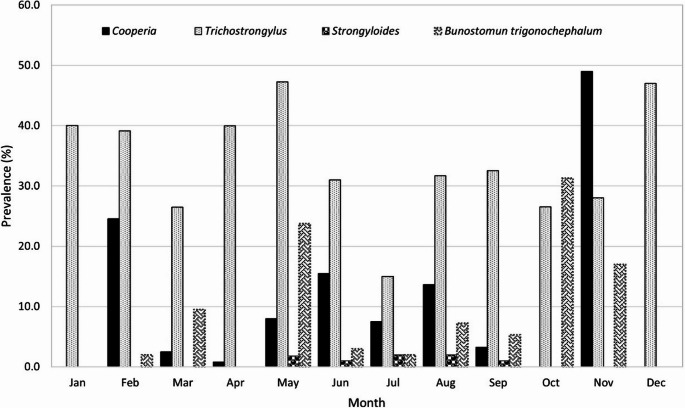
Prevalence of the main species of nematodes that parasitize the small intestine of ruminants in Mexico

In the monthly distribution it was noted that *Trichostrongylus* sp was reported in all months of the year with a prevalence higher than 20% except in July, while *Cooperia* sp had no records from October to January and its prevalence was less than 25% except in November when it almost reached 50% prevalence. *Bunostomum* also showed high prevalence in May and October and *Strongyloides* has been reported with a prevalence less than 3% (Fig. 3).

### Parasites of the cecum and colon

#### Oesophagostomum

In the colon, the genus *Oesophagostomum* Molin, 1861 has 47 species (Hodda 2022). In the literature is described *Oesophagostomum columbianum* in sheep, with a life cycle like other species such as *O. dentatum*,* O. quadrispinulatum*,* O. radiatum y O. venulosum* in domestic ruminants (Dash 1973). PCR identification has been required to characterize species of the subfamily Chabertiinae by means of the second internal transcribed spacer rDNA sequence (Newton et al. 1998). In Mexico several studies directed by Olivares (Olivares et al. 2001; Olivares-Orozco 2002) were carried out in 2000 and in 2019 they published the morphology of *O. columbianum* through observations with scanning electron microscopy (Olivares-Orozco and Gregorio Rodríguez-Diego 2019).

#### Trichuris

In the cecum, the genus *Trichuris* Roederer, 1761, has 107 species (Hodda 2022). This genus parasitizes the caecum of different hosts. The specific differentiation of the *Trichuris* genus has been controversial, for example, between *Trichuris trichiura* and *T. suis*. Morphological features may vary depending on environmental conditions and host-related factors, making species-level identification unreliable (García-Sánchez et al. 2019). The morphology of *T. suis*, *T. trichiura*, *T. colobae* y *T. ursinus* has been described for a long-time parasitizing primates and pigs. In sheep, the species *T. ovis* has been indicated (Gobind and Suresh 1954) and molecular differentiation has also been used with respect to *Trichuris discolor* (Vejl et al. 2017).

#### Prevalence of cecum and colon nematodes in Mexico

The main parasites of the cecum and colon indicated in the studies carried out in Mexico include *Oesophagostomum* and *Chabertia* in the colon and *Trichuris* in the cecum, identified in larvae and worms in slaughterhouses (Cuadro 7). The average prevalence was highest for *Oesophagostomum* (21.6%) and the highest value was observed in goats in slaughterhouses (44.5%). In the case of *Trichuris*, the highest value is reported in sheep feces identified as eggs and in the case of *Chabertia*, the highest prevalence is reported in larvae in goats (Table 7).

**Table 7:** Prevalence (%) of the three main genera of Gastrointestinal nematodes parasitic to the cecum and colon in ruminants in Mexico

Species and sample type	Oesophagostomum				Trichuris				Chabertia			
	*N*.	N2	Prevalence	SD	*N*.	N2	Prevalence	SD	*N*.	N2	Prevalence	SD
Buffalos												
Eggs/Larvae (Feces)						3	16.1	10.7				
Cattle												
Eggs/Larvae (Feces)	7	22	10.5	13.4	5	11	12.8	9.9	3	3	2.1	1.9
Slaughterhouse(worms)	1	1	19.2	.	1	1	2.4	.				
Goats												
Eggs/Larvae (Feces)	2	3	9.5	6.8	4	5	4.6	6.1	2	6	32.4	19.0
Slaughterhouse(worms)	1	4	44.5	26.1								
Sheep												
Eggs/Larvae (Feces)	9	18	22.6	27.7	4	4	25.1	13.1	3	3	3.6	3.8
Slaughterhouse(worms)	2	3	29.3	33.5	1	1	0.4	.				
Larvae (Pasture)	1	1	15.8	.	1	1	19.0	.	1	1	8.3	.
Average	23		**21.6**				**11.5**				**11.6**	

*N* Number or authors, *N2* number of records, *SD* Standard deviation

**Fig. 4:**
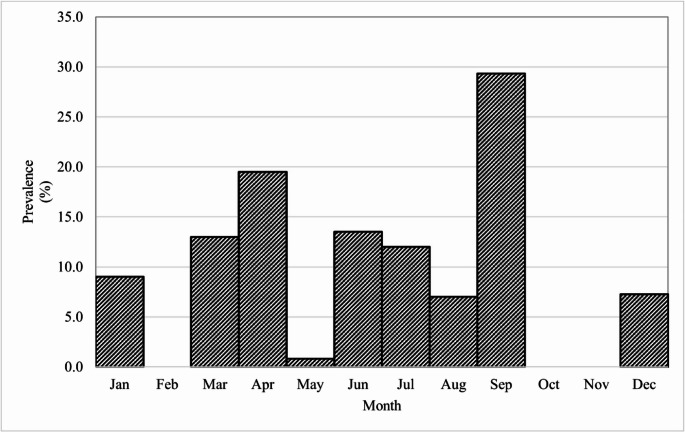
Monthly prevalence of *Oesophagostomum* in ruminants in Mexico

In the monthly distribution, *Oesophagostomum* showed a high prevalence in September while in February, October and November, no prevalence was found. In the case of *Trichuris* and *Chabertia* there were no references regarding prevalence by month (Fig. 4).

In addition to the most frequent genera of gastrointestinal parasites that have been reported in Mexico, the prevalence of other genera and species has been indicated, such as: in the peritoneum *Setaria cervi* (11%) in cattle (Mejía García and Orozco de Gortari 1987), *Nematodirus battus*, *N. spathiger*, *Muellerius capillaris* (George Sánchez and Quiroz Romero 1993), *Dictyocaulus filaria* (Nahed-Toral et al. 2003; Valladares-Carranza et al. 2024), *Mammomonogamus* spp. (Domínguez Alpízar et al. 1993), *Bunostomum phlebotomum* (Vásquez-Prats et al. 2004), B. *trigonocephalum* (González-Garduño et al. 2011), *Skrajabinema caprae* (Munguía-Xóchihua et al. 2018).

## Conclusions

According to the review described in this manuscript, it can be shown that the parasite with the highest prevalence is *Haemonchus contortus*, although other helminths such as *Moniezia* and *Oesophagostomum* are also indicated with high prevalence in Mexico. The high prevalence of gastrointestinal nematodes in ruminants makes it essential to consider control measures to reduce their prevalence. Although necropsy-based examinations remain valuable for diagnosing GIN, molecular tools, especially when applied to cultured larvae offer greater accuracy and diagnostic precision. However, we acknowledge the reviewer’s valuable point that the application of these molecular techniques can be limited in clinical and field settings by constraints of time and cost, particularly on extensive or small ruminant farms. In such cases, as we have discussed in previous work, a combination of rapid diagnostic methods and clinical observation remains a crucial and practical approach for the timely management of acute infections, such as haemonchosis.

## Data Availability

The datasets generated during and/or analysed during the current study are available from the corresponding author on reasonable request.
